# Clinical and Radiological Outcomes of Ultrasound-Guided Closed-Circuit Irrigation of Calcific Tendinitis of the Shoulder: A Prospective Study

**DOI:** 10.3390/life15081302

**Published:** 2025-08-16

**Authors:** Andrea De Grandis, Carlo D’Alessandro, Giovanni Sussan, Alberto Crimì, Lucrezia Tognolo, Daniele Coraci, Stefano Masiero, Roberto Ragazzi, Emilio Quaia, Filippo Crimì

**Affiliations:** 1 Institute of Radiology, Department of Medicine-DIMED, University of Padova, Via Giustiniani 2, 35128 Padova, Italy; andrea.degrandis96@gmail.com (A.D.G.); dottordalessandro@gmail.com (C.D.); giovanni.sussan@studenti.unipd.it (G.S.); emilio.quaia@unipd.it (E.Q.); 2 Orthopedics and Orthopedic Oncology Unit, Department of Surgery, Oncology and Gastroenterology DiSCOG, University of Padova, Via Giustiniani 2, 35128 Padova, Italy; alberto.crimi@aopd.veneto.it; 3 Rehabilitation Unit, Department of Neuroscience, University of Padova, 35122 Padova, Italy; lucrezia.tognolo@unipd.it (L.T.); daniele.coraci@unipd.it (D.C.); stef.masiero@unipd.it (S.M.); 4 Radiology Department, Ospedale dell’Angelo Mestre, ULSS 3 Serenissima, Via Paccagnella 11, 30174 Venezia, Italy; robertoragazzi1952@gmail.com

**Keywords:** closed-circuit irrigation, calcific tendinitis, pain management, ultrasound

## Abstract

Ultrasound-guided percutaneous interventions are well-established as effective treatments for shoulder calcific tendinopathy. In this work, we modified the conventional double-needle approach by incorporating a closed-circuit irrigation system and assessed the procedure’s clinical and radiological outcomes. We enrolled prospectively 24 patients (10 males; median age 54 years, IQR: 50–62) with painful calcific tendonitis of the shoulder between October 2023 and March 2024. All patients had a calcification > 5 mm treated with ultrasound-guided closed-circuit irrigation. Radiography, ultrasound evaluation, and OSS and SPADI clinical questionnaires were administered before the treatment and 3 months after. After the procedure, there was a significant reduction in the size of the calcifications (12 mm, IQR: 10–20 mm vs. 5.5 mm, IQR: 2–10 mm; *p* < 0.001). After the procedure, none of the patients experienced infections, while two developed bursitis. Three months after the procedure there were significant improvement in the OSS (16.5, IQR: 10–23 vs. 32, IQR: 36–45.5; *p* < 0.0001) and reduction in SPADI scores: pain (88, IQR: 74–95 before vs. 13, IQR: 4–24; *p* < 0.0001), disability (72, IQR: 60–90 before vs. 8, IQR: 4–20; *p* < 0.0001), and total score (78, IQR: 66–91 before vs. 11, IQR: 4–20; *p* < 0.0001). The closed-circuit double-needle irrigation for calcific tendinopathy of the shoulder is an effective treatment that improves both shoulder pain and function with a very low risk of short-term complications.

## 1. Introduction

Calcific tendinopathy of the shoulder is an acute or chronic painful condition caused by the deposit of calcium hydroxyapatite crystals inside or around the tendons of the rotator cuff [1,2]. Although this condition may present in different ways, its predominant characteristic is pain [1,2]. Patients may experience varying intensities of pain; acute cases are usually more painful, while chronic cases may present asymptomatic periods with relapsing–remitting patterns [1,2]. The presence of calcification alone is not enough to determine a painful shoulder condition. In fact, radiologically evident calcifications can be found in 7.5% to 20% of asymptomatic patients, while in patients with a painful shoulder, they may be found in 6.8% of cases [2,3]. Calcific tendinopathy of the shoulder is most predominant in people aged 30 to 60 years, particularly in females and those with sedentary jobs [2,3].

The most commonly affected muscle is the supraspinatus. Its tendon alone accounts for 63% of cases, while the combination of the supraspinatus and subscapularis tendons accounts for 20% of cases (subscapularis tendon alone 3%, infraspinatus tendon 7%, and subacromial bursa 7%) [4]. The disease progresses through three main stages: pre-calcific, calcific, and post-calcific [5,6].

The pre-calcific stage involves the transformation of tendon tissue into fibrocartilage, which facilitates calcium deposition. The calcific stage consists of a silent phase of calcium buildup followed by a resorptive phase characterized by intense pain. The post-calcific stage involves tendon remodeling and the potential for full recovery [5,6].

Radiographic and ultrasonographic classifications are helpful in identifying the type and stage of calcification [7,8]. There are two main X-Rays classifications for the calcific tendonitis of the shoulder: (i) the first one is the French Arthroscopic Society classification which subdivides calcifications into four categories from Type A, a sharply defined, homogeneous, and dense calcification to Type D, dystrophic calcification at the tendon insertion; (ii) the second is the Gartner and Heyer that includes three categories, from Type I, well-circumscribed, dense calcification to Type III, translucent and cloudy calcification with no clear circumscription [7,8].

In ultrasonography, calcifications are usually hyperechoic and may or may not display posterior acoustic shadowing. Their appearance can vary depending on the percentage of calcium present. In Bianchi and Martinoli’s classification, three different types of calcifications are described, corresponding to decreasing fractions of calcium deposition: Type I, hyperechoic foci with a well-defined acoustic shadow; Type II, hyperechoic foci with a mild acoustic shadow; and Type III, calcifications that appear almost isoechoic to the tendon, without an acoustic shadow. Type I is associated with the formative phase, while the resorptive phase is associated with Type II and Type III. The latter, often difficult to diagnose, is commonly described as fluid calcification [7,8].

Treatment approaches vary depending on symptom severity. Conservative management, including physical therapy and NSAIDs, is generally recommended for mild symptoms. Surgery is usually reserved as a last resort for more severe cases, due to its invasive nature [8,9,10]. Extracorporeal shockwave therapy is effective but not recommended during acute phases due to the potential for increased pain [9,11]. Ultrasound-guided percutaneous treatment is versatile and effective across different stages, with some exceptions for specific calcification types and locations (e.g., calcifications smaller than 5 mm, migrated into the subacromial bursa, or eroding the humeral cortex) [10].

The procedure aims to fragment, dissolve, and directly remove calcifications by injecting and aspirating a fluid, typically a saline solution. It can be performed using either one or two needles [12]. The double-needle technique creates a continuous in-and-out fluid flow, facilitating easier removal of calcium and maintaining fluid pressure within the calcification [13]. This helps prevent the peripheral rim from breaking and reduces the risk of calcium migrating to the bursa, which can cause post-procedural bursitis. While the single-needle approach is considered less invasive, the double-needle method may carry a higher risk of bleeding and infection [10]. To maintain sterility and avoid the need for refilling the syringe with saline solution after each flush, a closed irrigation circuit can be used during the double-needle procedure [14,15].

Although the use of a closed-circuit irrigation setup has been briefly described in technical notes and case series [14,15], the current literature lacks prospective studies systematically evaluating its clinical effectiveness in patients with calcific tendinopathy. To the best of our knowledge, this is the first prospective study designed to assess the clinical and radiological outcomes of the closed-circuit irrigation technique specifically for shoulder calcific tendonitis. The closed-circuit system may offer practical advantages over the standard double-needle technique, including reduced risk of contamination, increased procedural efficiency, and improved needle stability throughout the irrigation process.

Therefore, the aim of this paper was to evaluate the clinical and radiological effectiveness of the closed-circuit double-needle irrigation for calcific tendonitis of the shoulder.

## 2. Materials and Methods

### 2.1. Patients

After receiving approval from the local Ethical Committee (protocol CET-ACEV: 471n/AO/24), we prospectively enrolled patients with painful calcific tendonitis of the shoulder from October 2023 to March 2024.

This was a preliminary prospective study, and we assumed an improvement of 85% in the SPADI total score based on a review of the literature, which reported an improvement of 55% for the single- and double-needle methods together [12]. Using an alpha error of 0.05 and a power of 0.80, we calculated a required sample size of 18 patients. However, we included additional cases to account for potential patient dropouts during follow-up.

The inclusion criterion was the presence of a painful calcification of one of the rotator cuff tendons larger than or equal to 5 mm, documented on radiographic exam and confirmed with US examination, without bursal migration or humeral cortical erosion.

Exclusion criteria included refusal to participate in the study; concomitant rotator cuff tears in the same shoulder; or previous treatments with physical therapy, extracorporeal shockwave therapy, local steroid injections, or surgery.

The primary aim of the study was to assess the effect of pain and disability reduction with the ultrasound-guided closed-circuit irrigation system in patients affected by shoulder calcifications. Secondary aims were to i) evaluate the radiological (by means of US and X-Ray) reduction in calcifications’ dimensions in the tendons after the procedure; ii) record any complications due to the procedure; and iii) measure the time needed to perform the US-guided lavage.

### 2.2. Procedure

All procedures and ultrasound (US) examinations were performed using a Samsung RS85 (Samsung Medical, Seoul, Korea) with a linear 8–18 MHz probe, by a radiologist with 5 years’ experience in US-guided irrigation of calcific tendonitis of the shoulder (F.C.).

For the procedure, the patient was positioned supine, and the forearm was supinated. The skin was disinfected with three applications of iodopovidone disinfectant, which was then absorbed with sterile gauze to prevent skin irritation. To ensure complete sterility, the probe was disinfected, covered with a sterile sheath, and sterile gel was used. Initially, calcification was identified along its long and short axes. The entire procedure was performed under continuous ultrasound guidance using an in-plane approach. A 2% lidocaine solution was injected into the subcutaneous tissue, into the SASD bursa, and around calcification to provide local anesthesia.

**Figure 1:**
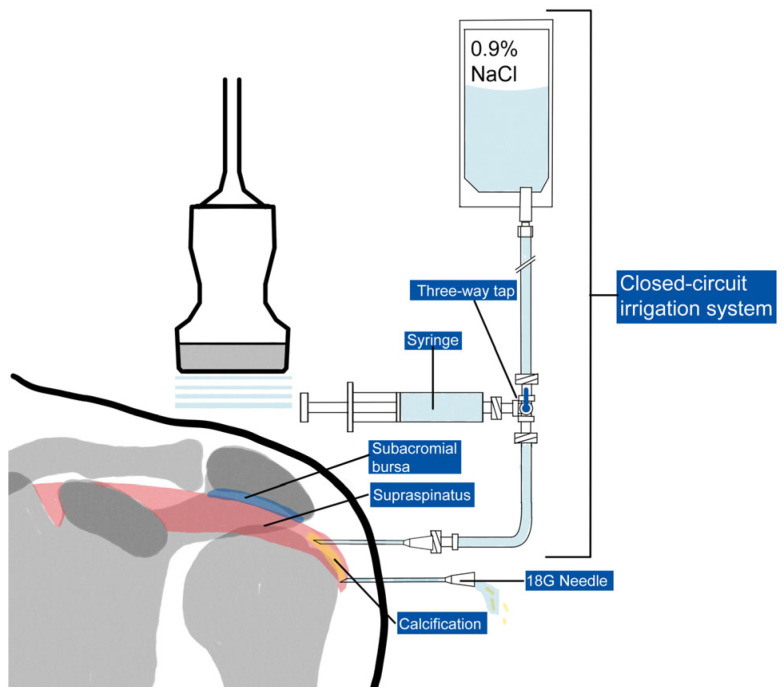
Schematic representation of the ultrasound-guided closed-circuit irrigation system for shoulder calcifications.

**Figure 2:**
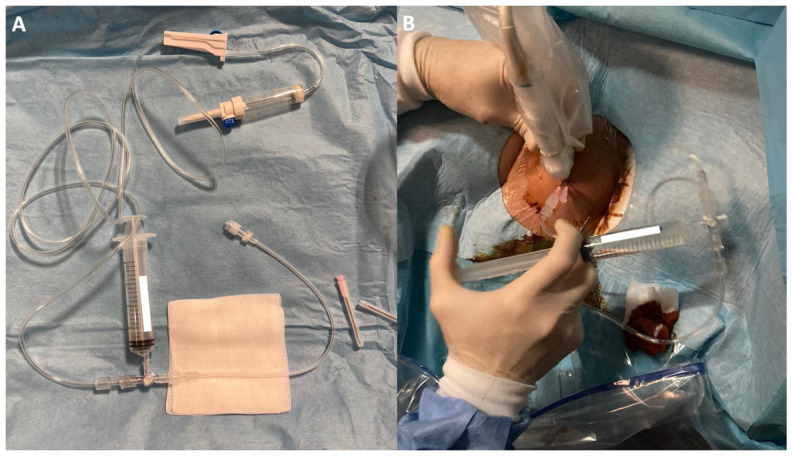
Ultrasound-guided closed-circuit irrigation of shoulder calcification: (**A**) Equipment: Warm sterile saline solution is connected via tubing to a three-way tap. A syringe is used to draw up the saline and direct its flow through an additional tube into one of the needles inserted into the calcification. (**B**) Procedure: The saline flush introduced through the first needle facilitates the evacuation of calcium deposits and saline solution through a second needle, which is not connected to the circuit.

Two 18G needles were then inserted with their tips into the calcification, positioned on the same acoustic plane, with the caudal needle inserted first to avoid reverberation artifacts. After insertion, a closed irrigation circuit was established. The circuit consisted of a warm (37°) sterile saline solution [16] connected via a tube to a three-way tap. A syringe was used to draw up the saline and flow it through another tube into one of the needles positioned in the calcification, causing calcium and saline solution to exit through the other needle not connected to the circuit (Figure 1 and Figure 2). This setup allowed for an almost continuous flow through the calcification and reduced the risk of needle displacement during syringe connection. The needles could be moved to reach the extremities of large calcifications and to scratch the walls, facilitating optimal calcium removal. In case of needle obstruction, the needles could be switched within the closed circuit, unblocking them and resuming irrigation. The material was collected by dripping into a sack provided with the sterile field kit.

At the end of the procedure, after at least 80% of calcium had been eliminated, one needle was removed, and the other was used to administer under US guidance into the SASD bursa 1 mL of methylprednisolone acetate (40 mg/mL Depo-Medrol; Pfizer Manufacturing Belgium, Puurs, Belgium) to provide post-procedural pain relief. Patients were also advised to use ice packs and take oral analgesics (acetaminophen).

The duration of each US-guided irrigation procedure with the closed irrigation circuit was measured in minutes, from the start of skin disinfection to post-treatment medications.

### 2.3. Imaging and Clinical Evaluation

All patients underwent radiographic and US evaluations before the treatment and 3 months afterward.

Shoulder ultrasound examinations were performed according to the technical guidelines of the European Society of Musculoskeletal Radiology (ESSR), examining the patient in a sitting position.

The largest diameter of the calcifications was measured before and after the procedure (on both radiographs and ultrasound, choosing the larger measurement for statistical analyses), and any post-procedural bursitis or other complications were noted at 3-month follow-up.

Two clinical questionnaires, the Oxford Shoulder Score (OSS) [17] and the Shoulder Pain and Disability Index (SPADI) [18], were administered to the patients before the procedure and 3 months after the treatment. These two questionnaires were designed to measure the pain and disability associated with shoulder conditions. All questionnaires were administered by clinicians, specifically radiology residents, in collaboration with orthopedic surgeons and physiatrists.

The OSS is a 12-item questionnaire that evaluates different dimensions of shoulder disorders, such as pain, ability to perform daily tasks, and overall joint function. Each question is scored from 0 (poorest outcome) to 4 (best outcome), giving a total possible score between 0 and 48. Higher totals reflect better shoulder performance and reduced pain. The SPADI includes 13 questions divided into two domains: pain (5 questions) and disability (8 questions). Responses are rated on a 0–10 scale, where 0 represents no pain or functional limitation, and 10 corresponds to the most severe pain or greatest difficulty in daily activities. Mean scores are calculated for each domain, and these can be combined to yield an overall score from 0 (optimal condition) to 100 (worst possible condition).

### 2.4. Statistical Analysis

Scientific Data are expressed as percentages, mean ± standard deviation (SD), or median and interquartile range (IQR), as appropriate. The Kolmogorov–Smirnov test was used to test the normality of the parameters. Since all the data did not follow a normal distribution, the median and IQR were used. For the comparison of clinical data, a paired *t*-test or Mann–Whitney test was used as appropriate. The OSS and SPADI scores before and after the procedures were measured and compared. The percentages of pain reduction after the procedures of our cohort and cohorts reported in the literature were compared using a two-sample *z*-test. The level of significance was set at *p* ≤ 0.01. Statistical analysis was performed using SPSS (version 29, IBM Corp., Armonk, NY, USA) and MedCalc (version 23.2.0, MedCalc Software, Ostend, Belgium).

## 3. Results

**Figure 3:**
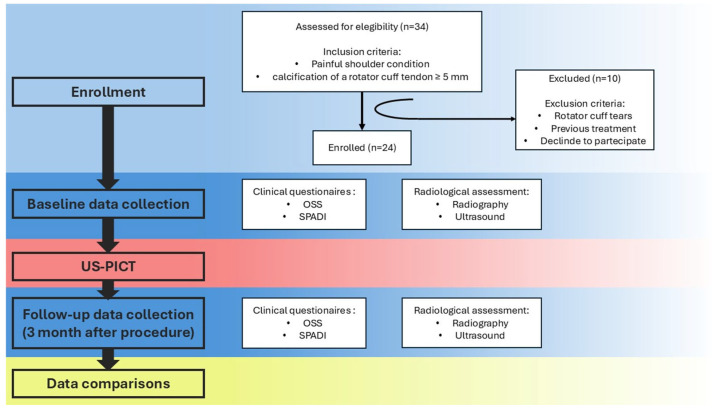
Study flowchart.

A total of 24 patients (10 males and 14 females) were enrolled in the study, as shown in Figure 3.

The characteristics of the patients are reported in Table 1.

**Figure 4:**
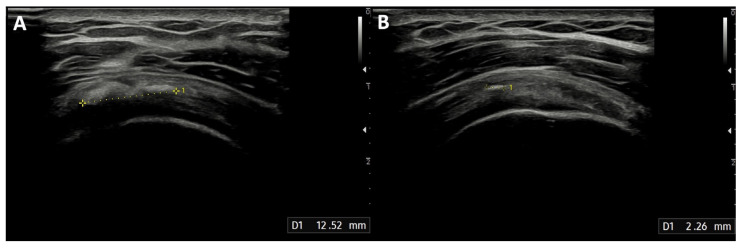
Reduction in supraspinatus tendon calcification treated with the ultrasound-guided closed-circuit irrigation procedure: (**A**) Ultrasound scan showing calcification of the supraspinatus tendon before the procedure, measuring 12.52 mm; thus, the patient was included in the study and underwent the closed-circuit double-needle irrigation technique. (**B**) Ultrasound image showing the same calcification 3 months after the procedure, with a significant reduction to a maximum diameter of 2.26 mm.

The median diameter of the calcification before the treatment was 12 mm (IQR: 10–20 mm). After the procedure, the calcification dimensions reduced significantly (Figure 4), with a median of 5.5 mm (IQR: 2–10 mm; *p* < 0.001), and 41 min (IQR: 39–45 min) was the median duration of the treatment. None of the patients experienced infections after the procedure. Two patients developed post-procedural bursitis, diagnosed with clinical and US examination 1 month after the procedure, which was easily resolved with a 4 mL injection of 2% lidocaine solution and steroids into the SASD bursa.

**Figure 5:**
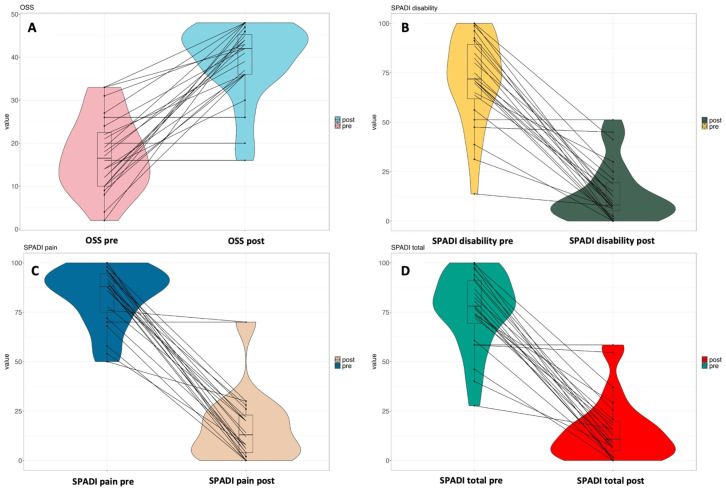
Violin plots showing the results of the questionnaires before and after the procedure: (**A**) Significant increase in the OSS (16.5, IQR 10–23 vs. 32, IQR 36–45.5; *p* < 0.0001); (**B**) significant reduction in the SPADI pain score (88, IQR 74–95 vs. 13, IQR 4–24; *p* < 0.0001); (**C**) significant reduction in the SPADI disability score (72, IQR 60–90 vs. 8, IQR 4–20; *p* < 0.0001); (**D**) significant reduction in the SPADI total score (78, IQR 66–91 vs. 11, IQR 4–20; *p* < 0.0001).

Regarding the clinical questionnaires, at diagnosis, the patients had a low median OSS of 16.5 (IQR: 10–23) and high SPADI scores: pain (88; IQR: 74–95), disability (72; IQR: 60–90), and total score (78; IQR: 66–91). As shown in Figure 5, the median OSS improved markedly following the procedure, increasing from 16.5 (IQR: 10–23) to 32 (IQR: 36–45.5; *p* < 0.0001). SPADI results showed substantial reductions across all domains: pain decreased from 88 (IQR: 74–95) to 13 (IQR: 4–24; *p* < 0.0001), disability from 72 (IQR: 60–90) to 8 (IQR: 4–20; *p* < 0.0001), and the total score from 78 (IQR: 66–91) to 11 (IQR: 4–20; *p* < 0.0001). The median improvement was 22.5 points (IQR: 14.5–31.5) for the OSS, while SPADI pain and disability improved by 68 (IQR: 59–85) and 62 points (IQR: 44–77), respectively, with an overall SPADI score improvement of 64 (IQR: 50–79).

The improvement we obtained in the SPADI total score (64%) was compared to the improvement in pain and disability reported in the literature review by Lanza et al. (2015) [12]. In this review, the mean improvement in the pain/disability score was 55% for US-guided irrigation of calcific tendonitis (60% of the studies using the single-needle technique and 40% with the double-needle technique) [12]. The difference between the 64% improvement in our study and the 55% reported by Lanza et al. was not statistically significant according to the two-sample *z*-test (*p* = 0.2891).

## 4. Discussion

The ultrasound-guided percutaneous treatment is a recognized and effective alternative to conservative management or surgery for calcific tendonitis of the shoulder [10].

In this study, we demonstrated that ultrasound-guided treatment using a closed-circuit irrigation system is effective for calcific tendinitis of the shoulder. This approach reduced the dimensions of the calcifications and improved patients’ pain and shoulder function. There were only two cases of post-procedural bursitis and no cases of infection, confirming the technical effectiveness and sterility of the procedure.

The total treatment duration, from the patient’s admission to discharge, was approximately 40 min, which is acceptable given the busy schedule of a radiology department.

Currently, two techniques are the most used for irrigation of calcific tendinitis of the shoulder, and which is the best is still debated. The first is the single-needle technique, which uses a needle inserted under ultrasound guidance into the calcification to gently push a flow of saline water into and out of the calcification with a syringe in order to progressively eliminate the calcium and resolve the calcification [10,11,12]. The second technique is the double-needle approach, in which the operator locates two needles inside the calcification under US guidance and discharges the saline solution inside one needle, allowing the water with calcium to come out from the other needle [10,11,12].

The latter technique enables a continuous flow of saline solution, which facilitates the removal of calcium and allows better control of the water pressure within the calcification, preventing rupture of the peripheral edge and reducing the risk of calcium migration into the SASD bursa [10]. On the other hand, the single-needle technique is considered less difficult even for non-expert radiologists and less invasive, since the double-needle technique can theoretically cause a greater risk of infection and bleeding by using two needles instead of one [10].

Until now, there have been no studies directly comparing the two techniques, so there is no evidence of the better performance of one compared to the other.

In order to maintain sterility and avoid the need to refill the syringe with saline solution after each flush, it has been recommended to use a closed irrigation circuit during the double-needle procedure [14,15].

However, to the best of our knowledge, there are no works in the literature that provide scientific data on the effectiveness of this variant of the double-needle technique in the treatment of calcific tendinopathy.

In our cohort of patients, we obtained a higher percentage of pain/disability improvement compared to what has been reported in a 2015 literature review by Lanza et al. [12], although not statistically significant (*p* = 0.2891), likely because of the limited number of patients enrolled in our cohort.

In the same review [12], a percentage of 10% minor complications was reported compared to the 2/24 (8%) minor complications of our series; hence, the rate was lower or at least similar to that reported in the literature.

Moreover, the technique allows the operator to keep the needles fixed in the same position throughout the procedure, avoiding displacement, as there is no need to reload the syringe with saline solution after each flush.

The increase in equipment cost compared to traditional methods is minimal, as the connection tubes are inexpensive.

The integration of a three-way stopcock connecting the saline reservoir, syringe, and the tubing leading to the needle enables efficient and aseptic syringe refilling without necessitating disconnection from the needle during saline replenishment. Furthermore, the continuous flow of saline facilitates more effective calcium clearance, thereby reducing the risk of needle lumen occlusion due to calcium deposition.

In clinical practice, this new technique could be considered, particularly for the treatment of larger calcifications or in patients at higher risk of infectious complications.

Some limitations of our study should be acknowledged. Firstly, it is a preliminary technical report involving 26 patients without a control arm. We are planning to conduct a prospective study comparing the closed-circuit irrigation technique with standard techniques. Additionally, the follow-up period of 3 months is relatively short, and a longer follow-up could provide more insights into the long-term efficacy of this procedure. Furthermore, the procedure was performed by a single expert radiologist, which could raise concerns about the reliability and reproducibility of the technique when carried out by less experienced clinicians. In future, prospective studies are needed comparing the closed-circuit irrigation technique with the standard single- and double-needle techniques, and a group of patients treated simply with intra-bursal steroid injection as the control group.

## 5. Conclusions

In conclusion, the proposed closed-circuit double-needle irrigation technique for calcific tendonitis of the shoulder has been proven to be an effective treatment that alleviates pain, enhances function, and carries a very low risk of short-term complications, offering results at least comparable to those reported for traditional techniques in the literature.

## Figures and Tables

**Table 1:** Patients’ characteristics: IQR = interquartile range.

Clinical Features	Data
Sex	10 (42%) males 14 (58%) females
Age	54 years (IQR: 50–62 years)
Shoulder subjected to calcification	10 (42%) right 14 (58%) left
Tendon involved in calcification	18 (75%) supraspinatus 4 (17%) subscapularis 2 (8%) infraspinatus
Calcification maximum diameter before treatment	12 mm (IQR: 10–20 mm)

## Data Availability

The data that support the findings of this study are available from the corresponding author upon reasonable request.
